# The role of psychosocial stress in the development of chronic musculoskeletal pain disorders: protocol for a systematic review and meta-analysis

**DOI:** 10.1186/s13643-017-0618-0

**Published:** 2017-11-03

**Authors:** Valentina Buscemi, Wei-Ju Chang, Matthew B. Liston, James H. McAuley, Siobhan Schabrun

**Affiliations:** 1 Brain Rehabilitation and Neuroplasticity Unit, School of Science and Health, Western Sydney University, Sydney, NSW Australia; 2 Neuroscience Research Australia (NeuRA), Sydney, NSW Australia

**Keywords:** Systematic review, Psychosocial stress, Musculoskeletal pain disorders, Prospective longitudinal studies

## Abstract

**Background:**

Psychosocial factors play an important role in chronic musculoskeletal pain disorders. Although psychosocial stress is likely to contribute to the development of chronic musculoskeletal pain, investigations are limited to work-related stress or examination of specific conditions such as upper limb pain. The purpose of this review is to assess the evidence for an aetiological role of psychological stress in chronic musculoskeletal pain disorders.

**Methods:**

A systematic review and meta-analysis will be conducted. Electronic databases will be searched using predefined search terms to identify relevant studies. Data will be extracted by two independent reviewers, and disagreement will be resolved by a third reviewer. Only prospective longitudinal studies that assess psychosocial stress at baseline will be included. The population of interest will be inception cohorts or cohorts of people who have not yet developed chronic musculoskeletal pain disorders. The primary outcome measure will be the onset of chronic musculoskeletal pain.

**Discussion:**

To our knowledge, this review will be the first to systematically explore the available evidence on the aetiological role of psychosocial stress for the development of chronic musculoskeletal pain disorders. This review has the capacity to inform clinical practice on the importance of an early identification and, consequently, treatment of individuals who present with acute musculoskeletal disorders accompanied by a high level of stress.

**Systematic review registration:**

PROSPERO CRD42017059949

**Electronic supplementary material:**

The online version of this article (10.1186/s13643-017-0618-0) contains supplementary material, which is available to authorized users.

## Background

Musculoskeletal disorders (MDs) are the second most common cause of disability worldwide, causing absence from work and increased costs for employers and the health care system. Disability rates due to chronic MDs rose by 45% between 1990 and 2010, and this figure is expected to increase further in future [1]. Although the term MDs indicates pains or aches of the musculoskeletal body system (muscles, joints, ligaments or tendons) [2], cognitive and psychosocial factors are also associated with MDs. Moreover, psychosocial factors have been shown to have a role in the transition to chronic low back pain. For example, observational longitudinal studies have shown that emotional distress (e.g. depression and anxiety) and maladaptive cognition (e.g. fear-avoidance and pain catastrophizing) negatively influence the progression of acute low back pain towards a chronic state [3, 4].

Another psychosocial factor, often studied in MDs, is psychosocial stress. Psychosocial stress is defined as a “perception of threat, with resulting discomfort, emotional tension, and difficulty in adjustment” [5: p.4]. Psychosocial stress can be triggered by different types of stressors, such as adverse life events (e.g. job loss or loss of a loved one), daily hassles (e.g. financial uncertainty or difficult relationships) or work-related stressors (e.g. low decision authority, job dissatisfaction or high job demands).

Work-related stress, occurring when work demand exceeds an individual’s knowledge and capacity to cope [6], has been explored extensively in different MDs and types of jobs. Studies have identified work-related stress as a significant risk factor for the development of chronic pain conditions [7–10]. In particular, a recent systematic review has shown that highly monotonous work and low social support are antecedents of MDs, with an OR (odds ratio) ranging from 1.15 to 1.6 [9]. In contrast, no systematic review has evaluated whether non-work-related psychosocial stress has an aetiological role in the development of chronic MDs. This review will be the first to systematically and critically evaluate the evidence on the causative role of non-work-related psychosocial stress in chronic MDs. A better understanding of the influence of psychosocial stress in chronic MDs is essential to facilitate treatments that target this aspect of a common and debilitating health problem.

## Methods

The following protocol has been written according to the MOOSE Guidelines for Meta-Analyses and Systematic Reviews of Observational Studies and the PRISMA-P (Preferred Reporting Items for Systematic Reviews and Meta-Analyses) guidelines [11, 12] [see Additional file 1]. The protocol has been registered at the International Prospective Register of Systematic Reviews (PROSPERO; registration number CRD42017059949).

### Review question

Does non-work-related psychosocial stress have an aetiological role in the development of chronic MDs in the general population?

In order to answer the review question, an association between exposure (psychosocial stress) and outcome (chronic musculoskeletal pain) will be sought. Specifically, a meta-analysis of the available scientific literature looking at the causal role (or aetiological role) of stress on pain outcome will be conducted. Only longitudinal prospective studies will be included, where stress is considered the exposure of interest, measured at baseline in an inception cohort, or a cohort that has not yet developed chronic pain. Adjustment for confounding factors (i.e. other psychological factors) will also be taken into account in the data extraction and analysis.

### Search strategy

Studies will be included if they are full-text articles, in press, accepted or published before the screening phase. The search will be carried out across the following databases: MEDLINE, Embase, PsychINFO, Pubmed, Scopus and CINAHL. The specific search strategy will be created by the project team with the support of a Health Science Librarian expert in systematic review searching. Only articles written in English will be included.

Keywords and Medical Subject Headings (MeSH) regarding stress and musculoskeletal pain will be used. Examples of the search terms include Stress*, mental fatigue, psycho* strain or burden or fatigue, demand*, pain, acute or chronic musculoskeletal pain, back pain, neck pain, upper and lower limb pain, cohort or longitudinal study. The combination of musculoskeletal pain, psychosocial stress and longitudinal studies search terms will be used in varying combinations to identify relevant literature. Search strategies will be customised to suit each database. An additional file shows a draft of the MEDLINE search strategy [see Additional file 2]. The reference lists of all relevant articles will be manually searched to identify any studies missed by electronic database searching.

### Participants

The study population will be adults (aged over 18 years) selected from the general population suffering from pain derived from any type of MD (e.g. back pain, neck pain, temporomandibular pain). Studies will be excluded if they investigate pain that is not of musculoskeletal origin, such as visceral or cancer pain or pain derived from central neurological conditions (e.g. stroke, spinal cord injury).

No restriction will be placed on participants’ gender. Since the aim of this study is to evaluate whether previous high levels of stress, or high stress present at the time of pain onset, have an aetiological role in the development of chronic MDs, studies will be included if participants are pain free at baseline, or if their pain, deriving from a MD, is acute (between 0 and 6 weeks from pain onset) [13].

### Types of studies

Studies will be accepted if (i) they are longitudinal observational studies measuring psychosocial stress at baseline and (ii) they use one or more scales or questionnaires to assess non-work-related psychosocial stress. Studies will be excluded if they (i) assess work-related stress, or use occupational stress models such as the Karasek demand-control model or the Siegrist effort-reward imbalance model [14, 15], (ii) assess distress and post-traumatic stress disorder (PTSD), as these are considered to relate to a specific psychological disorder, and (iii) are based on serious childhood events, such as assaults or maltreatment, as these are considered traumatic life events and may lead to serious mental health problems (e.g. PTSD).

### Outcomes

Eligible studies should report the development of chronic musculoskeletal pain, lasting 3 months or longer [13], as the primary outcome.

### Data management

The primary investigator will conduct the search strategy and remove duplicates using Endnote X7 (Thomson Reuters, USA). Studies obtained through the search strategy will be screened by two independent reviewers according to the inclusion and exclusion criteria. At the first stage, the reviewers will evaluate the title and abstract of each article and the full text of all eligible studies will be retrieved. If the reviewer is unsure about a study’s eligibility, a full-text evaluation will be conducted. Throughout data collection, disagreement will be resolved by consensus and, if unresolved, a third reviewer will be consulted. Included and excluded studies will be recorded in each screening phase.

### Data extraction

Two reviewers will independently conduct the following data extraction from each study using a pre-defined data extraction sheet [see Additional file 3]: (1) participant information (gender, sample size, drop-out rate, type of MD); (2) methods (study design, duration of follow-up); (3) type of scale or questionnaire used to assess stress; (4) strength of the association between the exposure of interest (non-work-related psychosocial stress) and the primary outcome, in terms of mean difference, odds ratio (OR), or relative risk (RR) and their 95% CI and (5) presence of comorbidities at baseline and/or follow up. Authors will be contacted a maximum of three times by e-mail if further information is needed. If the authors do not reply after the third attempt at contact, the data will be considered irretrievable.

### Assessment of methodological quality (risk of bias)

The methodological quality of the included studies will be assessed using a customised version of the Quality Assessment Tool for Observational Cohort studies and Cross-Sectional studies [16]. Two additional items from the QUIPS (Quality in Prognostic Studies) tool (Q6: Did the authors attempt to collect information on participants who dropped out? Q7: Are there important differences between participants who completed the study and those who did not?) [17] and two items considered important by the research team (whether loss to follow-up is accounted for in the analysis and whether the source of funding is provided) have been added to the risk of bias assessment tool [see Additional file 4]. Two independent reviewers will undertake the assessment of methodological quality. Any disagreement will be resolved by a third reviewer.

### Strategy for data synthesis

A quantitative synthesis is planned to pool data from included studies. Results from different stress-related questionnaires and single items, in case the entire questionnaire is not provided, will be aggregated to perform separate meta-analyses using RevMan (Version 5.3. Copenhagen: The Nordic Cochrane Centre, The Cochrane Collaboration, 2014). A minimum of two studies will be considered sufficient to perform a meta-analysis [18] and pool data using a random effects model.

The *χ*2 will be used to estimate heterogeneity between studies with a statistical significance of *p* ˂ 0.10. The *I* ^2^ will be used to identify the degree of heterogeneity, and *I* ^2^ ˃ 50% will be considered a substantial heterogeneity [19]. Effect estimates with 95% CIs will be presented.

### Subgroup analysis

If significant heterogeneity is found, a subgroup analysis will be conducted according to the type of pain condition (e.g. low back pain, neck pain) or type of psychosocial stress scale (e.g. Perceived Stress Scale).

### Sensitivity analysis

A score will be given to the included studies when assessing their methodological quality. The median value of the overall score will be used as a cut-off point to cluster the studies into low or high risk of bias groups. A further analysis will be conducted excluding studies with high risk of bias.

### Narrative analysis

If quantitative analysis is not possible, due to statistical or clinical heterogeneity, a narrative analysis will be undertaken. Narrative synthesis will be conducted based on levels of evidence used in previous systematic reviews [20]:

- Strong evidence: consistent findings from two or more high-quality cohorts.
- Moderate evidence: consistent findings from at least one high-quality study and one or more low-quality cohorts.
- Limited evidence: findings of one high-quality study or consistent findings in one or more low-quality studies.
- Conflicting evidence: inconsistent findings irrespective of study quality.
- No evidence: no studies found.

### Publication bias

In order to investigate the introduction of publication bias, a random effects version of Egger’s test will be utilised and visualised through a funnel plot [21]. The minimum number of articles suggested to examine publication bias is ten [22].

## Discussion

To our knowledge, this review will be the first to systematically explore the evidence available on the aetiological role of non-work-related psychosocial stress in the development of chronic MDs. This review has the capacity to inform clinical practice regarding the importance of early assessment, identification and treatment of patients with MDs who are also experiencing high levels of stress. Indeed, stress can be considered a modifiable factor that, if assessed and promptly recognised, can be addressed and potentially prevent the development of chronic pain. This study should help practitioners to become more aware of the effect of stress on pain, and, on the importance of working alongside mental health clinicians in order to minimise stress in people with acute musculoskeletal disorders.

Finally, a better understanding of the role of psychosocial stress in the development of chronic MDs should facilitate further research in the area of prevention and early interventions, through stress management programs for those who present with high levels of stress at the onset of their MD.

### Limitations

As data will be extracted using only full-text articles and excluding data from grey literature, potential publication bias may be introduced.

### Ethics and dissemination

This systematic review does not require ethical approval, as only a secondary analysis of data already available in scientific databases will be conducted. The results of this review will be submitted for peer-reviewed publication and will be presented at relevant conferences.

### Review status

The reviewers have commenced searching relevant studies on the electronic databases. This review is expected to be complete by September 2017.

## Additional files

Additional file 1: PRISMA-P (Preferred Reporting Items for Systematic review and Meta-Analysis Protocols) 2015 checklist. Recommended items to address in a systematic review protocol. (DOCX 15 kb)

Additional file 2: MEDLINE search strategy. MEDLINE search strategy that will be used to conduct the proposed systematic review. (DOCX 12 kb)

Additional file 3: Data extraction sheet. Pre-defined data extraction sheet that will be used to extract data of the included studies. (DOCX 14 kb)

Additional file 4: Checklist to assess methodological quality of studies. Checklist that will be used to assess the included studies. (DOCX 16 kb)

## Abbreviations

MD: Musculoskeletal disorders
MOOSE: Meta-analysis of Observational Studies in Epidemiology
OR: Odds ratio
PRISMA-P: Preferred Reporting Items for Systematic Reviews and Meta-analyses
PTSD: Post-traumatic stress disorder
