# Supplementary material for: The role of psychosocial stress in the development of chronic musculoskeletal pain disorders: protocol for a systematic review and meta-analysis
Source: Syst Rev. 2017 Nov 3;6:224. doi: 10.1186/s13643-017-0618-0 (PMC5670509; doi:10.1186/s13643-017-0618-0)
Supplement: Supplementary file 2 — MEDLINE search strategy. MEDLINE search strategy that will be used to conduct the proposed systematic review. (DOCX 12 kb) [file 13643_2017_618_MOESM2_ESM.docx]

**Additional file 2. MEDLINE search strategy**

1. burden.mp.

2. psycho* distress.mp.

3. exp burnout/

4. demand*.mp.

5. exp mental load/ or exp mental stress/ or mental strain.mp.

6. caregiver burden/ or psycho* burden.mp.

7. exp emotional stress/

8. mental suffering.mp.

9. mental strain.mp.

10. psycho* exertion.mp.

11. mental fatigue.mp.

12. psycho* strain.mp.

13. mental stress/ or family stress/ or stress*.mp. or emotional stress/ or life stress/ or social stress/ or school stress/ or environmental stress/

14. cohort.mp. or exp risk factor/

15. predictor.mp. or exp prediction/

16. longitudinal.mp. or exp longitudinal study/

17. 14 or 15 or 16

18. musculoskeletal pain/ or myofascial pain/ or jaw pain/ or heel pain/ or hip pain/ or ankle pain/ or shoulder pain/ or limb pain/ or low back pain/ or complex regional pain syndrome/ or hand pain/ or foot pain/ or spinal pain/ or pain.mp. or neck pain/ or leg pain/ or pain/ or face pain/ or musculoskeletal chest pain/ or chronic pain/

19. 1 or 2 or 3 or 4 or 5 or 6 or 7 or 8 or 9 or 10 or 11 or 12 or 13

20. 17 and 18 and 19
