# Supplementary material for: The role of psychosocial stress in the development of chronic musculoskeletal pain disorders: protocol for a systematic review and meta-analysis
Source: Syst Rev. 2017 Nov 3;6:224. doi: 10.1186/s13643-017-0618-0 (PMC5670509; doi:10.1186/s13643-017-0618-0)
Supplement: Supplementary file 3 — Data extraction sheet. Pre-defined data extraction sheet that will be used to extract data of the included studies. (DOCX 14 kb) [file 13643_2017_618_MOESM3_ESM.docx]

| **Authors**  **(year)** | **Participants** | | | | **Methods** | | **Exposure of interest** | | **Comorbidities** | |
| --- | --- | --- | --- | --- | --- | --- | --- | --- | --- | --- |
|  | Gender | Sample size | Drop-out rate | Baseline pain condition | Study design | Duration of follow-up | Stress-related scale or questionnaire | MD, OR, RR (95% CI) | Yes/No | Type |
|  |  |  |  |  |  |  |  |  |  |  |
|  |  |  |  |  |  |  |  |  |  |  |
|  |  |  |  |  |  |  |  |  |  |  |
|  |  |  |  |  |  |  |  |  |  |  |
|  |  |  |  |  |  |  |  |  |  |  |
|  |  |  |  |  |  |  |  |  |  |  |
|  |  |  |  |  |  |  |  |  |  |  |
|  |  |  |  |  |  |  |  |  |  |  |
|  |  |  |  |  |  |  |  |  |  |  |
|  |  |  |  |  |  |  |  |  |  |  |
|  |  |  |  |  |  |  |  |  |  |  |
|  |  |  |  |  |  |  |  |  |  |  |
|  |  |  |  |  |  |  |  |  |  |  |
|  |  |  |  |  |  |  |  |  |  |  |
|  |  |  |  |  |  |  |  |  |  |  |
|  |  |  |  |  |  |  |  |  |  |  |

**Additional file 3. Data extraction sheet**
