# Supplementary material for: The role of psychosocial stress in the development of chronic musculoskeletal pain disorders: protocol for a systematic review and meta-analysis
Source: Syst Rev. 2017 Nov 3;6:224. doi: 10.1186/s13643-017-0618-0 (PMC5670509; doi:10.1186/s13643-017-0618-0)
Supplement: Supplementary file 4 — Checklist to assess methodological quality of studies. Checklist that will be used to assess the included studies. (DOCX 16 kb) [file 13643_2017_618_MOESM4_ESM.docx]

**Additional file 4. Checklist to assess methodological quality of studies**

| **Criteria** | **Yes** | **No** | **Other (CD, NR, NA)*** |
| --- | --- | --- | --- |
| **1. Was the research question or objective in this paper clearly stated?** |  |  |  |
| **2. Was the study population clearly specified and defined?** |  |  |  |
| **3. Was the participation rate of eligible persons at least 50%?** |  |  |  |
| **4. Were all the subjects selected or recruited from the same or similar populations (including the same time period)?** |  |  |  |
| **5. Were inclusion and exclusion criteria for being in the study pre-specified and applied uniformly to all participants?** |  |  |  |
| **6. Did the authors attempt to collect information on participants who dropped out?** |  |  |  |
| **7. Are there important differences between participants who completed the study and those who did not?** |  |  |  |
| **8. Was a sample size justification, power description, or variance and effect estimates provided?** |  |  |  |
| **9. For the analyses in this paper, were the exposure(s) of interest measured prior to the outcome(s) being measured?** |  |  |  |
| **10. Were losses of patients to follow-up taken into account in the analysis?** |  |  |  |
| **11. Was the timeframe sufficient so that one could reasonably expect to see an association between exposure and outcome if it existed?** |  |  |  |
| **12. For exposures that can vary in amount or level, did the study examine different levels of the exposure as related to the outcome (e.g., categories of exposure, or exposure measured as continuous variable)?** |  |  |  |
| **13. Were the exposure measures (independent variables) clearly defined, valid, reliable, and implemented consistently across all study participants?** |  |  |  |
| **14. Was the exposure(s) assessed more than once over time?** |  |  |  |
| **15. Were the outcome measures (dependent variables) clearly defined, valid, reliable, and implemented consistently across all study participants?** |  |  |  |
| **16. Were the outcome assessors blinded to the exposure status of participants?** |  |  |  |
| **17. Was loss to follow-up after baseline 20% or less?** |  |  |  |
| **18. Were key potential confounding variables measured and adjusted statistically for their impact on the relationship between exposure(s) and outcome(s)?** |  |  |  |
| **19. Was the source of funding provided?** |  |  |  |
